# Quantum blockchain based on asymmetric quantum encryption and a stake vote consensus algorithm

**DOI:** 10.1038/s41598-022-12412-0

**Published:** 2022-05-21

**Authors:** Wusheng Wang, Yang Yu, Lingjie Du

**Affiliations:** School of Physics and National Laboratory of Solid State Microstructures, Nanjing University, Nanjing, 210093 China

**Keywords:** Information theory and computation, Quantum physics

## Abstract

As emerging next-generation information technologies, blockchains have unique advantages in information transparency and transaction security. They have attracted great attentions in social and financial fields. However, the rapid development of quantum computation and the impending realization of quantum supremacy have had significant impacts on the advantages of traditional blockchain based on traditional cryptography. Here, we propose a blockchain algorithm based on asymmetric quantum encryption and a stake vote consensus algorithm. The algorithm combines a consensus algorithm based on the delegated proof of stake with node behaviour and Borda count (DPoSB) and quantum digital signature technology based on quantum state computational distinguishability with a fully flipped permutation (${\text{QSC}}{\text{D}}_{\text{ff}}$) problem. DPoSB is used to generate blocks by voting, while the quantum signature applies quantum one-way functions to guarantee the security of transactions. The analysis shows that this combination offers better protection than other existing quantum-resistant blockchains. The combination can effectively resist the threat of quantum computation on blockchain technology and provide a new platform to ensure the security of blockchain.

## Introduction

The concept^1^ of blockchain technology was first introduced by Satoshi Nakamoto in 2008. Blockchain is a decentralized block of data linked in a chronological chain network to provide a distributed shared ledger and database. For example, in the first blockchain system, i.e. Bitcoin, each block contains two parts, namely, the block header and block body. The block header contains the hash value of the current block, the hash value of the previous block, the timestamp, and information about the Merkel tree; the block body contains the transaction information and the corresponding digital signature. One advantage of the blockchain is the usage of a distributed network, which provides the transparency and security of transaction information. After more than ten years of rapid development, this technology is not limited to Bitcoin and other cryptocurrencies but also attracts intense attention from multidisciplinary areas, such as finance, energy, medical care, and government affairs.

At the core of blockchain technologies, the most important aspects are consensus algorithms and digital signatures. Consensus algorithms can be used to generate blocks, while digital signatures can secure transaction information. For example, the consensus algorithm used in the Bitcoin network is proof of work (PoW)^1^, which allows every miner to compete through computing power based on a hash algorithm. The miner with higher hash power tends to have larger probabilities to find the correct hash solution, and the first miner that finds the correct hash value will generate a new block. In addition, there are other consensus algorithms such as proof of stack (PoS)^2^, delegated proof of stack (DPoS)^3^, and delegated proof of stake with node’s behaviour and Borda count(DPoSB)^4^. They do not rely on computing power and thus could lower the power consumption. There is also a Byzantine algorithm^5^ that achieves consensus in communication in the presence of malicious nodes.

Digital signatures are an essential application of public-key cryptography. Encryption methods commonly used in the digital signatures of a classical blockchain are Rivest-Shamir-Adleman (RSA)^6^ and elliptic curve cryptography (ECC)^7^. These well-developed encryption algorithms are too complex for classical computers to crack, ensuring the security of the digital signatures. However, Shor and others have found that a quantum algorithm can effectively solve the integer decomposition problem and the discrete logarithmic problem^8^, which are the critical parts of the encryption methods. In this case, the security of blockchain technology based on the digital signatures is under the threat of quantum computation.

Several physical systems have been developed to realize quantum computation. Quantum supremacy was demonstrated on a programmable superconducting quantum processor with 53 qubits by Google^9^. Pogorelov et al.^10^ performed 50-qubit ion trap quantum computing. Moreover, Zhong et al.^11^ demonstrated a 76-qubit quantum computer with photons for boson sampling and a programmable quantum nanophotonic chip with many photons^12^.

Therefore, it has become urgent to develop new methods to protect against the threat of quantum computing. One effective approach is to develop quantum cryptography techniques based on the unique nature of quantum physics. For example, the quantum signature technology based on quantum state computational distinguishability with fully flipped permutations (${\text{QSC}}{\text{D}}_{\text{ff}}$) problem, utilizing the complexity of ${\text{QSC}}{\text{D}}_{\text{ff}}$ problem for quantum computation, can guarantee the security of the signature process. In addition, there are also quantum key distribution (QKD) techniques used in quantum information, such as the most famous BB84 protocol^13^. These techniques help to improve security in communication processes even in the presence of quantum computation.

In this case, these algorithms can be involved in blockchain technologies, which further improve system securities. Several attempts have been made. For example, quantum key distribution (QKD) techniques, such as the most famous BB84 protocol^13^, used in quantum information have been applied to blockchains^14^; quantum entanglement in time has been used to produce blocks^15^, which is combined with quantum signature algorithms^16^. However, quantum signatures are not used in the QKD blockchain algorithm; a blockchain generated by the use of entanglement in time cannot trace back the transaction information, and thus the improvement in the overall security of the blockchain is poor.

To guarantee blockchain network security under quantum supremacy, we propose a quantum blockchain method that combines the DPoSB consensus algorithm^4^ and quantum signatures established with quantum signature technology based on quantum state computational distinguishability with a fully flipped permutation (${\text{QSC}}{\text{D}}_{\text{ff}}$) problem^17^. The former is developed from DPoS, which keeps the voting system and considers the influence of malicious behaviours in votes to improve security when malicious nodes are in a blockchain system. A quantum signature method using a quantum asymmetric cryptography approach is a signature method designed based on the complexity of the ${\text{QSC}}{\text{D}}_{\text{ff}}$ problem for quantum computation to guarantee the security of the signature process. Here we combine them together. The blockchain generates blocks by DPoSB and signs transactions by a quantum one-way function^18^ based on the ${\text{QSC}}{\text{D}}_{\text{ff}}$ problem. Mining here is not necessary to make great savings on computing resources, which greatly saves computing resources and increases the speed of block generation. Different from other quantum signature methods^14,15,16^, this method is not constrained^19,20^ by probabilities and does not require a large number of one-time pads, which thus saves substantial communication overheads. Discussions about security models and quantum information-theoretical security are introduced in the security analysis. It can be found that our blockchain is secure even in the malicious adversary model. Our results show that this signature method in quantum blockchain is more secure than other quantum signatures. In this paper, the data structure of blockchain network is introduced in “Data structure of the blockchain ” and our quantum blockchain section algorithm is analyzed in “Quantum blockchain algorithm” section. Then, the security of the blockchain algorithm is analyzed in “Security analysis of the blockchain” section, and the blockchain algorithm is compared with other existing quantum blockchains in “Comparison with other quantum blockchain signature methods” section. The conclusion is given at the end.

## Data structure of the blockchain

**Figure 1:**
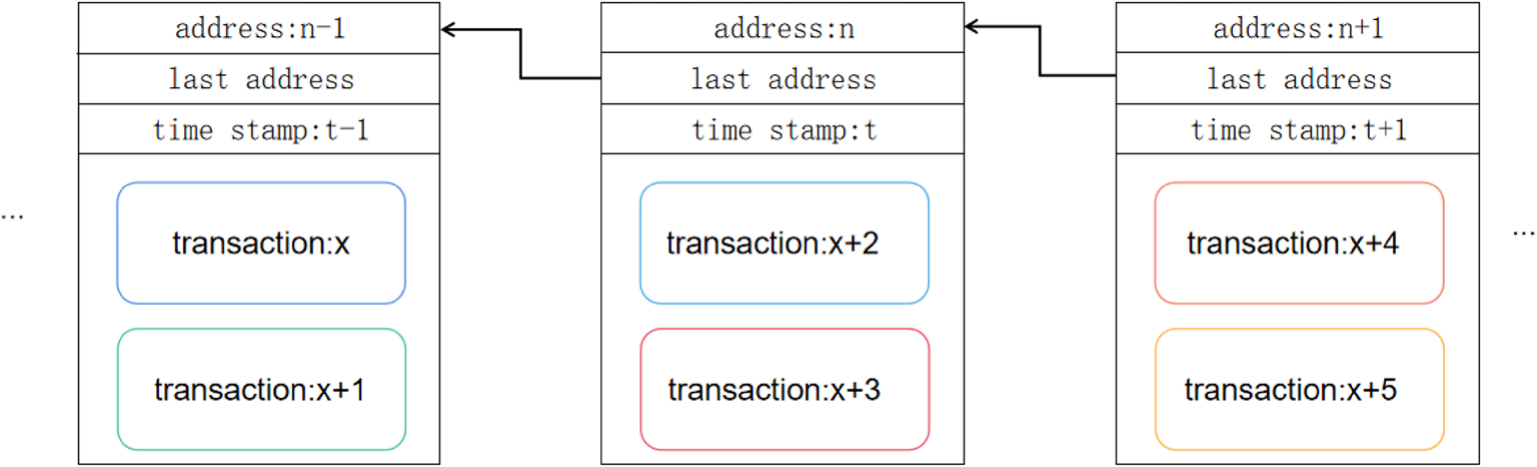
The data structure of the quantum blockchain. The block header contains the address of the current block, the address of the previous block and the timestamp. The block body contains the transaction that has passed through the quantum signature verification process. The arrows between two blocks indicate that we can find one block according to its next block.

A block acting as a unit in our blockchain system is constructed by a block header and a block body, as shown in Fig. 1. The information in the block header contains the address of the current block, the address of the previous block and the timestamp. The block body contains the transaction information that has passed through the quantum signature verification process. Due to the vital point of DPoSB, blockchain nodes do not need to participate in mining; namely, there is no computing force competition; thus, the hash value in the block is not necessary and can be replaced with the explicit address. We can begin from the block in the end to find the desired information according to the block addresses.

## Quantum blockchain algorithm

In the blockchain, the signer generates the transaction and then uses a private key to sign, and the receiver authenticates the transaction by using the signer’s public key to ensure transaction security in the aspect of cryptography.

First, our quantum blockchain network contains $N$ nodes, and n ($N$>2n) witness nodes are elected to generate blocks in turn by DPoSB^13^. Then the nodes sign transactions through a quantum one-way function based on the ${\text{QSC}}{\text{D}}_{\text{ff}}$ problem. The witness nodes verify the transactions signed by the nodes and package the transactions into blockchain network if it passes through the verification process.

### Blocks created by DPoSB

One key characteristic of DPoSB is voting, which is developed from DPoS. By the application of voting, the computing source originally used for mining can be largely saved. In voting, the n nodes with the highest votes are elected as the witness nodes responsible to generate blocks in turn. Let us assume that there are $N$ nodes in a blockchain system. First, 2n ($N$>2n) candidate nodes are elected by voting, and then n witness nodes among the candidate nodes are elected. However, sometimes there are some malicious nodes appearing in the system, which hinder the generation of blocks.

There are four types of malicious behaviours denoted by $r$. Each $r$ is distributed by a weight ${Q}_{r}$ and the maximum threshold ${T}_{r}$ is the largest number of times the behaviour $r$ is accepted in the system. Below are the types of $r$,

$r$=1(fp): This indicates that the failure of transaction package, where ${Q}_{1}=0.4$ and ${T}_{1}$=Max1.

$r$=2 (fv): This indicates that the failure of block check, where ${Q}_{2}=0.3$ and ${T}_{2}$=Max2.

$r$=3 (bn): The failure of node communication, where ${Q}_{3}=0.2$ and ${T}_{3}$=Max3.

$r$=4 (other): Other types of malicious behaviour, where ${Q}_{4}=0.1$ and ${T}_{4}$=Max4.

Then DPoSB introduces malicious behaviour punishment calculation in the algorithm to address this issue and the mechanism of the Borda score to fairly select the witness nodes. We calculate the malicious behaviour weight ratio ${N}_{i}^{Bw}$ for the $i$ th node:

$$N_{i}^{Bw} = \mathop \sum \limits_{r = 1}^{B} \left( {\frac{{t_{ir} }}{{T_{r} }} \times Q_{r} } \right),\left( {0 \le t_{ir} \le M_{r} ,0 \le i \le N,0 \le r \le 4} \right),$$

where ${t}_{ir}$ represents the number of times the behaviour $r$ is performed by the $i$ th node makes.

The valid vote to define the $i$ th node is:

$$V_{i} = \left( {\mathop \sum \limits_{j}^{N} P_{j}^{\left( t \right)} } \right) \times \left( {1 - N_{i}^{Bw} } \right),\left( {0 \le i \le N,0 \le j \le N,0 \le t} \right),$$

where ${P}_{j}^{(t)}$ indicates the number of votes by $j$ th node for $i$ th node in round $t$ of block generation (all participants produce a block once as the end of one round).

Then, we sort the valid votes for all nodes, and 2n nodes with the highest votes are elected as the candidate nodes.

The next step is to select n witness nodes from these candidate nodes. We construct the preference matrix:

$$\left[ {\begin{array}{llll} {r_{11}^{k} } & {r_{12}^{k} } & \cdots & {r_{1m}^{k} } \\ {r_{21}^{k} } & {r_{22}^{k} } & \cdots & {r_{2m}^{k} } \\ \ldots & \ldots & \cdots & \ldots \\ {r_{m1}^{k} } & {r_{m2}^{k} } & \cdots & {r_{mm}^{k} } \\ \end{array} } \right],$$

where $${ }r_{ij}^{k} = \left\{ {\begin{array}{l} {1,{\text{voter k prefers }}x_{i} \succ x_{j} } \\ {0,{\text{voter k does not prefer }}x_{i} \succ x_{j} } \\ \end{array} } \right.$$.

Then we have the $k$ th node’s preference value for the $i$ th candidate node: ${r}_{i}^{k}={\sum }_{j=1}^{N}{r}_{ij}^{k}$ and obtain the Borda score matrix:

$$\left[ {\begin{array}{*{20}c} {r_{1}^{1} } & {r_{1}^{2} } & \cdots & {r_{1}^{N} } \\ {r_{2}^{1} } & {r_{2}^{2} } & \cdots & {r_{2}^{N} } \\ \cdots & \cdots & \cdots & \cdots \\ {r_{C}^{1} } & {r_{C}^{2} } & \cdots & {r_{C}^{N} } \\ \end{array} } \right].$$

We calculate the cumulative Borda scores for each candidate node: ${r}_{i}={\sum }_{k=1}^{N}{r}_{i}^{k}$.

The Borda scores are sorted for all candidate nodes, and the n candidate nodes with the highest scores are elected as the witness nodes.

**Figure 2:**
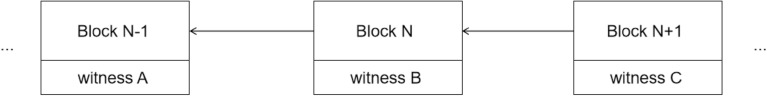
Witness nodes generate block in turn, and every witness node generates one block in one round.

The witness nodes can generate blocks in turn, as shown in Fig. 2.

### Transaction signing and verification process

Then the nodes sign transactions through a quantum one-way function based on the quantum state computational distinguishability with fully flipped permutations QSCD_ff_problem.

In quantum algorithms, quantum gate operations ^21^ can be performed on qubits, which include Hadamard ($H$), qubit flip ($X$), phase flip ($Z$) operations. The quantum state of a single qubit can be represented as $|\mathrm{\varphi }\rangle =\mathrm{sin\theta }|0\rangle +\mathrm{cos\theta }{\mathrm{e}}^{\mathrm{ia}}|1\rangle$, where $|0\rangle$ and $|1\rangle$ are the counterparts of 0 and 1 in the classical computation. A quantum gate operation can be represented as performing a unitary operator $\mathrm{U}$ on the quantum state, $\mathrm{U}|\mathrm{\varphi }\rangle$, to produce a target quantum state.

In the quantum algorithm, signing and verification processes are necessary to ensure a transaction. Here, we use the quantum one-way function based on the ${\text{QSC}}{\text{D}}_{\text{ff}}$ problem to finish the signing process.

#### A brief introduction to the ${\text{QSC}}{\text{D}}_{\text{ff}}$ question

We define ${N}_{*}$= {$n\in N$, $n$ is even and $n$/2 is odd}. For each $n\in {N}_{*}$, ${S}_{n}$ is used to represent a symmetric group of degree $n$. Then we use ${\kappa }_{n}$= {$\pi \in {S}_{n}$: ${\pi }^{2}$=$id$ and $\forall i\in \{\mathrm{1,2},\cdots ,n\}$ [$\pi (i)\ne i$], where $id$ represents all the identity permutations. Each $\pi$ can be represented as an odd permutation that is the product of $n$/2 disjoint transposition ^22^.

Then we have $\left|{\kappa }_{n}\right|=\frac{n!}{{\left(\sqrt{2}\right)}^{n}}$ and the following definition:

For each $\pi \in {\kappa }_{n}$, there are quantum states ${\rho }_{\pi }^{+}(n)$ and ${\rho }_{\pi }^{-}(n)$:

$${\rho }_{\pi }^{+}\left(n\right)=\frac{1}{2n!}{\sum }_{\sigma \in {S}_{n}}\left(\left|\sigma \right.\rangle +\left|\sigma \pi \right.\rangle \right)\left(\left.\langle \sigma \right|+\left.\langle \sigma \pi \right|\right),$$

$$\rho_{\pi }^{ - } \left( n \right) = \frac{1}{2n!}\mathop \sum \limits_{{\sigma \in S_{n} }} \left( {\left. {\left| \sigma \right.} \right\rangle - \left| {\left. {\sigma \pi } \right\rangle } \right.} \right)\left( {\left\langle \sigma \right. - \left\langle {\sigma \pi } \right.} \right).$$

For a symmetric group of degree $n$, each group element can be represented as an arrangement with $n$ elements, such as a group element $(\mathrm{1,2},3)$ in ${S}_{3}$, which can be represented as quantum states: $\left|1\right.\rangle \left|10\right.\rangle \left|0\right.\rangle$.

The ${\text{QSC}}{\text{D}}_{\text{ff}}$ problem is to distinguish the following two quantum states for each $n\in {N}_{*}$:${\rho }_{\pi }^{+}(n{)}^{\otimes P(n)},{\rho }_{\pi }^{-}(n{)}^{\otimes P(n)}$, where the $P(n)$ represents a polynomial.

Ref.^22^ has proven that if $\pi$ $\in$ ${\kappa }_{n}$ is random and unknown there is no quantum algorithm that can solve the ${\text{QSC}}{\text{D}}_{\text{ff}}$ problem with non-negligible advantage. However, this problem can be quickly solved with the solution of $\pi$ so that $\pi$ would serve as a trapdoor in the quantum signatures.

##### A distinguishing algorithm for the ${\text{QSC}}{\text{D}}_{\text{ff}}$ problem

**Figure 3:**
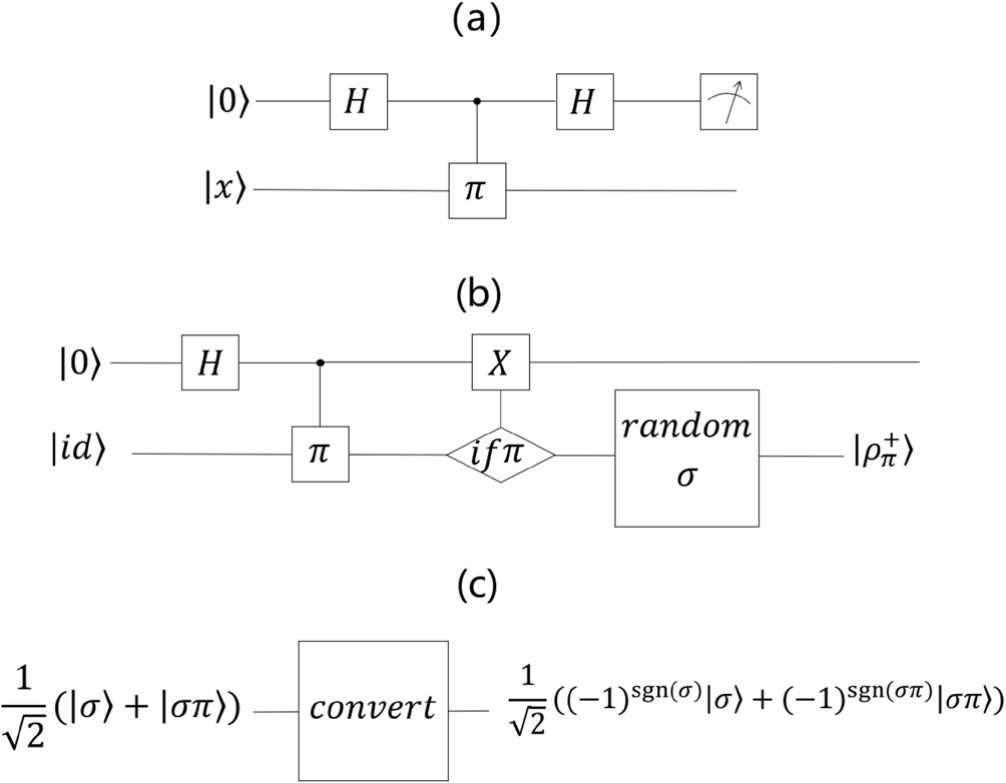
(**a**) The quantum circuit of the distinguishing algorithm, where *H* represents the Hadamard operations $H\left|0\right.\rangle =\frac{1}{\sqrt{2}}\left(\left|0\right.\rangle +\left|1\right.\rangle \right)=\left|+\right.\rangle$ and $H\left|1\right.\rangle =\frac{1}{\sqrt{2}}\left(\left|0\right.\rangle -\left|1\right.\rangle \right)=\left|-\right.\rangle$ and $\pi$ represents the $\pi$ operation $\pi \left|\sigma \right.\rangle =\left|\sigma \pi \right.\rangle$. We perform the quantum circuit from left to right. (**b**) The quantum circuit of the ${\rho }_{\pi }^{+}(n)$ generation algorithm, where “if $\pi$” means “if we read $\left|\pi \right.\rangle$ from this register” and “random $\sigma$” means “perform a random permutation $\sigma$ on this register”. (**c**) The quantum circuit of the conversion algorithm, where we perform the following “convert” operation: $convert[\frac{1}{\sqrt{2}}\left(\left|\sigma \right.\rangle +\left|\sigma \pi \right.\rangle \right)]=\frac{1}{\sqrt{2}}\left({\left(-1\right)}^{\mathit{sgn}(\sigma )}\left|\sigma \right.\rangle +{\left(-1\right)}^{\mathit{sgn}(\sigma \pi )}\left|\sigma \pi \right.\rangle \right)$.

*Step 1* The quantum circuit used here is shown in Fig. 3a. For a quantum state $x$, $x$ ∈ {${\rho }_{\pi }^{+}(n)$, ${\rho }_{\pi }^{-}(n)$}, we prepare the initial state $\left|0\right.\rangle \left|x\right.\rangle$. $\left|0\right.\rangle$ is input into the first register (a device used to preserve one or more quantum states) of the quantum circuit, and $\left|x\right.\rangle$ is input into the second register. The Hadamard operation ($H$) is performed on $\left|0\right.\rangle$, to obtain:

$$H\left|0\right.\rangle =\frac{1}{\sqrt{2}}\left(\left|0\right.\rangle +\left|1\right.\rangle \right)=\left|+\right.\rangle ,H\left|1\right.\rangle =\frac{1}{\sqrt{2}}\left(\left|0\right.\rangle -\left|1\right.\rangle \right)=\left|-\right.\rangle.$$

*Step 2* The ${C}_{\pi }$ operation is performed on the second register controlled by the first register to obtain:

$${C}_{\pi }\left|0\right.\rangle \left|\sigma \right.\rangle =\left|0\right.\rangle \left|\sigma \right.\rangle , {C}_{\pi }\left|1\right.\rangle \left|\sigma \right.\rangle =\left|1\right.\rangle \left|\sigma \pi \right.\rangle .$$

*Step 3* The $H$ operations is performed on the first register.

*Step 4* The $Z$ measurement is performed on the first register, and $x={\rho }_{\pi }^{+}(n)$ if $\left|0\right.\rangle$ is obtained, otherwise, $x={\rho }_{\pi }^{-}(n)$.

##### ${{\varvec{\rho}}}_{{\varvec{\pi}}}^{+}({\varvec{n}})$ generating algorithm

${\rho }_{\pi }^{+}(n)$ can be generated by the following steps. The quantum circuit is shown in Fig. 3b.

*Step 1* We prepare the quantum state $\left|0\right.\rangle \left|id\right.\rangle$, input $\left|0\right.\rangle$ into the first register, and input $\left|id\right.\rangle$ into the second register. Then, we perform the $H$ operation on the first register, and obtain $\left|+\right.\rangle$ and the $\left|id\right.\rangle$.

*Step 2* The ${C}_{\pi }$ operation is performed on the second register and controlled by the first register.

*Step 3* If the second register reads $\left|\pi \right.\rangle$, we perform a qubit flip operation ($X$) ^21^ on the first register.

*Step 4* A uniformly random permutation $\sigma$ is performed on the second register.

*Step 5* The final state of the second register is output.

##### Converting algorithm

The symbol function $sgn(\cdot )$ on the symmetric group ${S}_{n}$ is as follows:

If σ is an even permutation, $\mathit{sgn}(\sigma )=0$; if $\sigma$ is an odd permutation, $\mathit{sgn}(\sigma )=1$.

We can convert ${\rho }_{\pi }^{+}(n)$ to ${\rho }_{\pi }^{-}(n)$ by the following operation:

$$\frac{1}{\sqrt{2}}\left(\left|\sigma \right.\rangle +\left|\sigma \pi \right.\rangle \right)\to \frac{1}{\sqrt{2}}\left({\left(-1\right)}^{\mathit{sgn}(\sigma )}\left|\sigma \right.\rangle +{\left(-1\right)}^{\mathit{sgn}(\sigma \pi )}\left|\sigma \pi \right.\rangle \right)$$

(even permutation × odd permutation = odd permutation, odd permutation × odd permutation = even permutation; $\left|\pi \right.\rangle$ is an odd permutation.).

The quantum circuit is shown in Fig. 3c.

#### Signing transaction process

Below we use an example to show the detailed processes. Alice serves as a signer and Bob as a verifier. Jack acts as the private key generator (PKG), which is a trusted node in the blockchain system, and never exposes the signer's private key or imitates the signer to sign messages. Alice is ready to send a transaction message that she encodes as a bit string $TA({m}_{1},{m}_{2},\cdots ,{m}_{n})$, ${m}_{i}\in \{\mathrm{0,1}\}$. The transaction can be signed by following steps ^17^.

##### Key generation phase

*Step 1* Alice randomly selects an odd permutation $\pi \in {\kappa }_{n}$ as the private key, where $n$ is the length of the bit string. Then, the unconditionally secure deterministic secure quantum communication (DSQC) protocol^23^ is used to write the private key in the blockchain to secretly share it. In this case, Jack secretly holds $(ID,\pi )$ pair, where $ID$ is Alice's identity code.

**Figure 4:**
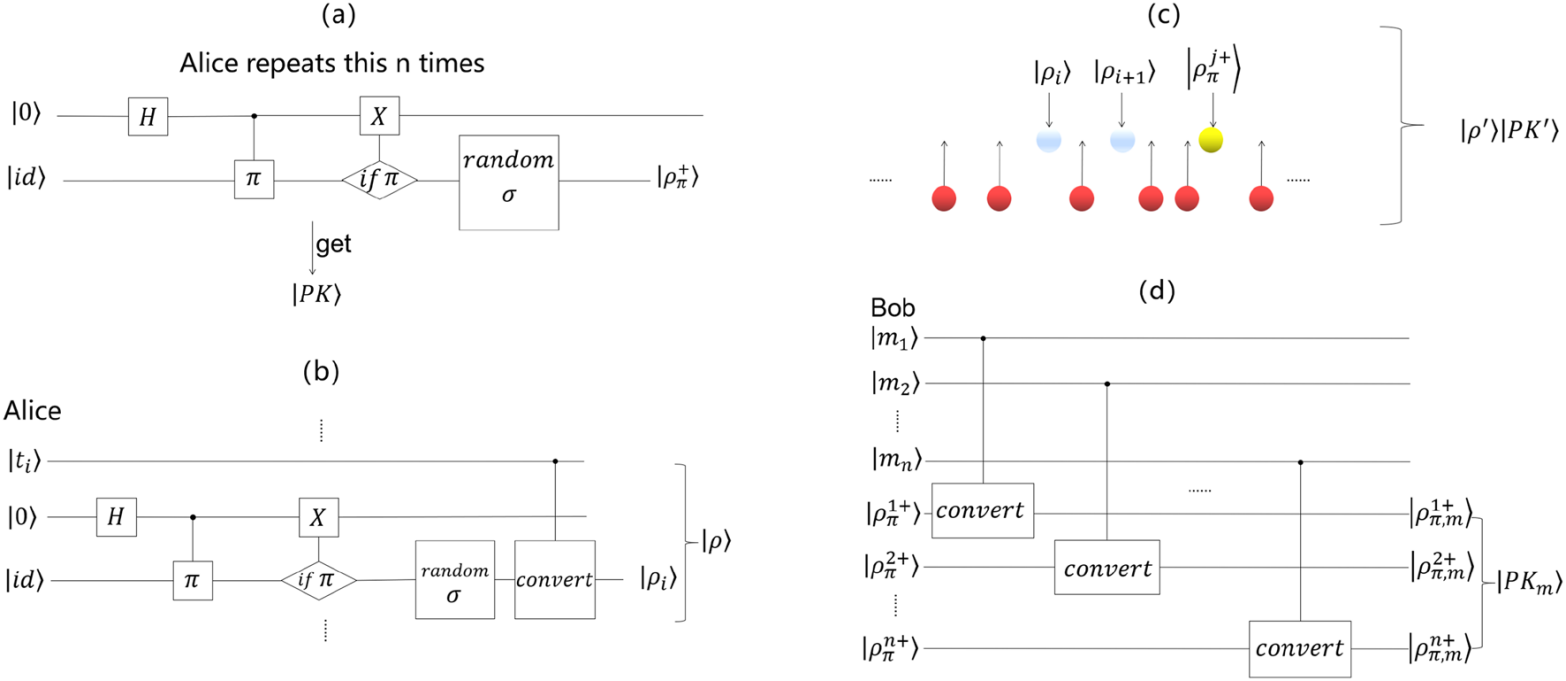
(**a**) Alice repeats the ${\rho }_{\pi }^{+}(n)$ generation algorithm n times with her private key to obtain the public key, where the H, “if $\pi$” and “random $\sigma$” operations are the same as the operations in Fig. 3. (**b**) Alice uses this quantum circuit to obtain encrypted sequence $\left|\rho \right.\rangle$, where we perform a “C-convert” operation: if $\left|{t}_{i}\right.\rangle =1$, we perform the “convert” operation shown in Fig. 3c; if $\left|{t}_{i}\right.\rangle =0$, we do not perform any operation. (**c**) The red balls represent decoy particles, the white balls represent encrypted sequence $\left|\rho \right.\rangle$, and the yellow balls represent public key $\left|PK\right.\rangle$. Alice inserts decoy particles randomly into $\left|\rho \right.\rangle \left|PK\right.\rangle$ and obtains the sequence $|{\rho }^{{^{\prime}}}\rangle |P{K}^{{^{\prime}}}\rangle$ to check for eavesdropping. (**d**) Bob uses this quantum circuit to obtain $|{PK}_{m}\rangle$, where every “C-convert” operation is the same as the “C-convert” operation in (**b**).

*Step 2* Alice performs the ${\rho }_{\pi }^{+}(n)$ generation algorithm to obtain the public key $\left|PK\right.\rangle ={\otimes }_{i=1}^{n}{\rho }_{\pi }^{i+}$. (One bit one key), as shown in Fig. 4a.

*Step 3* Alice has a key pair $(\left|PK\right.\rangle ,\pi )$.

##### Signing phase

*Step 1* Alice performs a permutation $\pi$ operation on $TA$ and obtains the bit string $t$: $\pi (TA)=t$, where the $t=({t}_{1},{t}_{2},\cdots ,{t}_{n})$, ${t}_{i}\in \{\mathrm{0,1}\}.$

*Step 2* Through the conversion algorithm, Alice encrypts $t$ as a quantum sequence: $\rho ={\otimes }_{i=1}^{n}{\rho }_{i}$, where $${\rho }_{i}=\left\{\begin{array}{c}{\rho }_{\pi }^{+}(n), {\text{i}}{\text{f}} \, {t}_{i}=0\\ {\rho }_{\pi }^{-}(n), {\text{i}}{\text{f}} \, {t}_{i}=1\end{array}\right.$$, as shown in Fig. 4b.

*Step 3* Alice prepares $r$ decoy particles ($r\gg 2n$_)_, which are distributed randomly in $(\left|1\right.\rangle , \left|0\right.\rangle ,\left|+\right.\rangle ,\left|-\right.\rangle )$. She inserts $r$ decoy particles randomly into $\left|\rho \right.\rangle \left|PK\right.\rangle$ and gets the sequence $\left|\rho {^{\prime}}\right.\rangle \left|PK{^{\prime}}\right.\rangle$ to check eavesdropping^8^, as shown in Fig. 4c. She then sends $\{TA,ID,\left|\rho {^{\prime}}\right.\rangle ,\left|PK{^{\prime}}\right.\rangle \}$ to Bob.

*Step 4* After receiving $\{TA,ID,\left|\rho {^{\prime}}\right.\rangle ,\left|PK{^{\prime}}\right.\rangle \}$, Alice exposes the location of the decoy particles. Bob checks the particles with the corresponding base. If there is no error, Bob takes the next step, and otherwise the signature generation phase is restarted.

*Step 5* Bob performs an eavesdropping check, drops all the decoy particles and finally holds $\{TA,ID,\left|\rho \right.\rangle ,\left|PK\right.\rangle \}$ as the quantum signature of Alice.

##### Verifying phase

*Step 1* Bob converts the public key $|PK\rangle$ to $|{PK}_{m}\rangle$ based on $TA$, where $\left|P{K}_{m}\right.\rangle ={\otimes }_{i=1}^{n}{\rho }_{\pi ,m}^{i}$ and $${\rho }_{\pi ,m}^{i}=\left\{\begin{array}{c}{\rho }_{\pi }^{+}(n),{\text{i}}{\text{f}} \, {m}_{i}=0\\ {\rho }_{\pi }^{-}(n),{\text{i}}{\text{f}} \, {m}_{i}=1\end{array}\right.$$, as shown in Fig. 4d.

**Figure 5:**
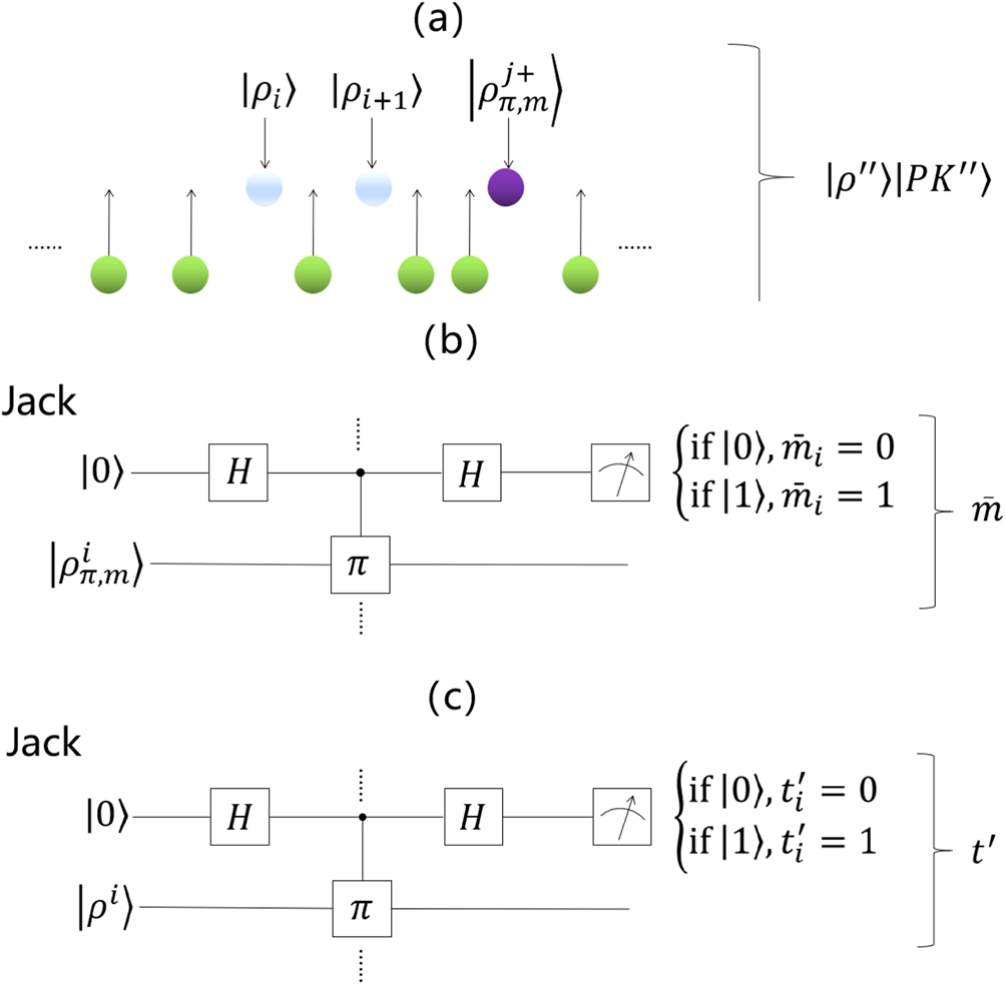
(**a**) The green balls represent decoy particles, the white balls represent encrypted sequence $\left|\rho \right.\rangle$, and the purple balls represent public key $\left|PK\right.\rangle$. Bob randomly inserts the decoy particles into $\left|\rho \right.\rangle \left|P{K}_{m}\right.\rangle$ to obtain the sequence $|{\rho }^{{^{\prime}}{^{\prime}}}\rangle |P{K}^{{^{\prime}}{^{\prime}}}\rangle$ to check for eavesdropping. (**b**) Jack uses this quantum circuit to obtain bit string $\overline{m }$. For every ${\rho }_{\pi ,m}^{i}$, if $\left|0\right.\rangle$ is read from the first register, then ${\bar{m}}_{i}=0$; if $\left|1\right.\rangle$ is read from the first register, then ${\bar{m}}_{i}=1$. (**c**) Jack uses this quantum circuit to obtain a bit string $t{^{\prime}}$. For every ${\rho }_{i}$, if $\left|0\right.\rangle$ is read from the first register, then ${\overline{t} }_{i}=0$; if $\left|1\right.\rangle$ is read from the first register, then ${\overline{t} }_{i}=1$.

*Step 2*:Bob prepares the $r$ decoy particles ($r\gg 2n$_)_, which are randomly in $(\left|1\right.\rangle ,\left|0\right.\rangle ,\left|+\right.\rangle ,\left|-\right.\rangle )$. He randomly inserts the $r$ decoy particles into $\left|\rho \right.\rangle \left|P{K}_{m}\right.\rangle$ to get the sequence $\left|\rho {^{\prime}}{^{\prime}}\right.\rangle \left|PK{^{\prime}}{^{\prime}}\right.\rangle$ to check for eavesdropping, as shown in Fig. 5a. He then sends $\{ID,\left|\rho {^{\prime}}{^{\prime}}\right.\rangle ,\left|PK{^{\prime}}{^{\prime}}\right.\rangle \}$ to Jack.

*Step 3* After Jack receives $\{ID,\left|\rho {^{\prime}}{^{\prime}}\right.\rangle ,\left|PK{^{\prime}}{^{\prime}}\right.\rangle \}$, Bob exposes the location of the decoy particles. Jack checks the particles with the corresponding base. If there is no error, Jack takes the next step, and otherwise the signature generation phase is restarted.

*Step 4* Jack discards all decoy particles and recovers $\left|\rho {^{\prime}}{^{\prime}}\right.\rangle \left|PK{^{\prime}}{^{\prime}}\right.\rangle$ to $\left|\rho \right.\rangle \left|P{K}_{m}\right.\rangle$.

*Step 5* Jack recovers the private key $\pi$ according to the identity code $ID$, and obtains the bit string $\overline{m }$ by distinguishing ${\rho }_{\pi ,m}^{i}$, where $${\bar{m}}_{i}=\left\{\begin{array}{c}0,{\rho }_{\pi ,m}^{i}={\rho }_{\pi }^{+}(n)\\ 1,{\rho }_{\pi ,m}^{i}={\rho }_{\pi }^{-}(n)\end{array}\right.$$, as shown in Fig. 5b. Then, permutation $\pi$ is performed on $\overline{m }$, and $\overline{t }=\pi (\overline{m })$ is obtained.

*Step 6* Jack distinguishes ${\rho }_{i}$, and obtains the bit string $t{^{\prime}}$, where $$t{^{\prime}}=\left\{\begin{array}{c}0, if {\rho }_{i}={\rho }_{\pi }^{+}(n)\\ 1, if {\rho }_{i}={\rho }_{\pi }^{-}(n)\end{array}\right.$$, as shown in Fig. 5 (c). If ${\overline{t} }_{i}$ = $t{^{\prime}}$, Jack claims validation and Bob accepts the signature.

#### Package the transaction into blockchain

**Figure 6:**
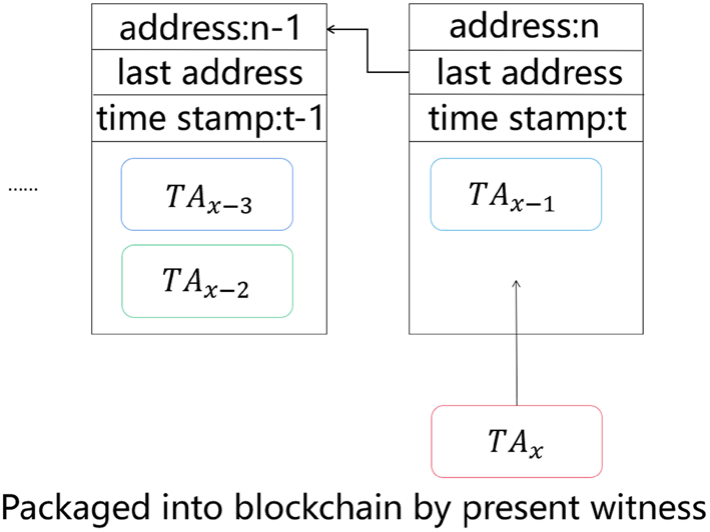
Transactions verified by 2/3 of witnesses can be packaged into a blockchain by the present witness, where the data structure of the blocks is the same as the data structure in Fig. 1, and $T{A}_{x}$ is a valid transaction that needs to be packaged.

In actual applications, the witness codes elected under DPoSB should be considered trusted signature verifiers. After more than 2/3 of the witness nodes accept the signature, the generated transaction information $TA$ is valid and packed into the block generated by the current witness node, as shown in Fig. 6. However, when the verifying phase is completed, if less than 2/3 of the witness nodes accept the signature, $TA$ is discarded by the current witness node.

## Security analysis of the blockchain

### Security model

Before reviewing the security of our blockchain, we would explain two security models used in information theory and cryptography^24^.

*Semi-honest adversary model* Suppose there are some semi-honest adversaries in a system and they follow a protocol correctly but may keep some necessary information to infer additional information later.

*Malicious adversary model* Suppose there are some malicious adversaries in a system and they may not only keep necessary information to infer additional information, but also attempt to perform breaking-protocol malicious behaviours to get additional information.

In the block generation process, a semi-honest adversary can only keep public information of the block header and block body. Then he cannot infer any useful additional information, because there are no secrets in the public information. In the signing process, a semi-honest adversary can attempt to infer the private key of a signer (the only secret), which however cannot work as shown in "Security of private keys" Section. Therefore our blockchain can keep security in the semi-honest adversary model.

We will demonstrate the security in the malicious adversary model in the next three sections. Generally, when an algorithm or a protocol can keep security in the malicious adversary model it is safer.

### Security of the generation of blocks

Consensus algorithms are used in the generation of blocks, and different consensus algorithms have distinct security levels. There are three main breaking-protocol attacks in this process, which belong to the malicious adversary model: 1. Double-spending attacks^1^. 2. Attacks that crack the hash value in a short time^14^. 3. Nodes that disturb the generation of blocks on purpose^13^. Then we will explain how our blockchain has robustness in the block generation process to these attacks in the malicious adversary model.

Attacker nodes can forge another blockchain secretly to forge information in blocks, which is defined as double-spending attacks. The success rate of this attack is higher when the computing force is larger. The success rate becomes 100% when the computing force of one node is larger than half of the total computing force of the blockchain system. However, this attack can be defended against in our blockchain algorithm because it is based on the computing force that is not needed in our algorithm.

An attack that cracks the hash value in a short time is a special attack based on a quantum computer. The quantum computer can use quadratic acceleration to crack the hash value through the Grover algorithm^25^, which makes nodes that have quantum computers dominate the blockchain systems. However, this attack is still based on computing force, so it can be defended in our blockchain algorithm.

As shown in "Blocks created by DPoSB" Section, in blockchain systems, some nodes may intentionally disturb the generation of blocks. In the DPoSB algorithm, malicious behaviours can be recorded by blockchain systems, and these records have impacts on the nodes’ scores during the elections. Because the chance that a node is elected as a witness is smaller when it has more malicious behaviours, our blockchain algorithm can also defend against this attack.

### Quantum information-theoretical security

In quantum asymmetric encryption, an encryption has quantum information-theoretical security if the quantum cyphertexts have computational indistinguishability^26^.

We can claim that two quantum ensembles ${\rho }_{1}$ and ${\rho }_{2}$ are computationally indistinguishable, if for every probabilistic polynomial algorithm $A$, every positive polynomial $P(.)$ and sufficiently large positive integer $n$ the following inequation can be satisfied ^26^:

$$|{P}_{r}(A({\rho }_{1})=1)-{P}_{r}(A({\rho }_{2})=1)|<\frac{1}{P(n)},$$

where ${P}_{r}(.)$ represents the probability.

In our blockchain algorithm, the cyphertexts are ${\rho }_{\pi }^{+}(n)$ and ${\rho }_{\pi }^{-}(n)$. Then we define that ${\rho }_{1}={\rho }_{\pi }^{+}(n{)}^{\otimes P(n)}$, ${\rho }_{2}={\rho }_{\pi }^{-}(n{)}^{\otimes P(n)}$ and need to prove:

$$|{P}_{r}(A({\rho }_{\pi }^{+}(n{)}^{\otimes P(n)})=1)-{P}_{r}(A({\rho }_{\pi }^{-}(n{)}^{\otimes P(n)})=1)|<\frac{1}{P(n)}.$$

Assume that we have a probabilistic polynomial algorithm ${A}_{l}$, which makes:

$$|{P}_{r}({A}_{l}({\rho }_{\pi }^{+}(n{)}^{\otimes P(n)})=1)-{P}_{r}({A}_{l}({\rho }_{\pi }^{-}(n{)}^{\otimes P(n)})=1)|\ge \frac{1}{P(n)}.$$

It means we have an efficient algorithm to distinguish signature cyphertexts ${\rho }_{\pi }^{+}(n)$ from ${\rho }_{\pi }^{-}(n)$ efficiently, corresponding to solving the $\rm QSC{D}_{ff}$ problem. However, according to the hardness of the $\rm QSC{D}_{ff}$ problem as proved in ref.^22^, the problem cannot be solved in polynomial time. Thus, it can be claimed that our blockchain has quantum information-theoretical security.

### Security of the signing process

The malicious attacks which can be used in this process are eavesdropping, forging, repudiation and interception. Then we will explain how our blockchain can have robustness in the signing process to these attacks in the malicious adversary model.

#### Security of private keys

The security of private keys should be assured in two ways.

First, it has been proven that no quantum algorithm can crack the private keys of signers in polynomial time when there is no private key $\pi$ ^22^ because one cannot distinguish signature cyphertexts ${\rho }_{\pi }^{+}(n)$ from ${\rho }_{\pi }^{-}(n)$ efficiently, as discussed in "Quantum information-theoretical security" Section.

Second, because private keys are selected from ${\kappa }_{n}$ and $|{\kappa }_{n}|=\frac{n!}{{\sqrt{2}}^{n}}$, the attacker only has a chance of $\frac{{\sqrt{2}}^{n}}{n!}$ to obtain the private keys (note that the divergence of $n!$ is far stronger than ${\sqrt{2}}^{n}$). In this case, the success rate of brute attacks is sufficiently small, which means that the success rate of signatures randomly generated by attackers is negligible.

#### Security against eavesdropping

As mentioned above, we can use the BB84^13^ protocol to defend against eavesdropping. Because of the particularity of quantum states, eavesdropping can result in the collapses of quantum states and destroy the decoy states. By the second checkout process in BB84, the verifier could determine if there is any eavesdropping through the measurement of decoy states. In addition, eavesdropping by cloning signatures is not possible because of the quantum no-cloning theorem^21^.

#### Security against forging

There are two forging attack approaches. The first is forging signatures by using the transaction information of signers, and the second is forging the transaction information of signers.

In the first approach, a signer generates transaction information $TA$ and public key $\left|PK\right.\rangle$ and then uses the private key to generate signature $|{\rho }_{1}\rangle$. An attacker wants to forge a signature with $TA$ and the signer’s private key, which makes $|{\rho }_{1}\rangle \ne |{\rho }_{2}\rangle$. According to the signature algorithm mentioned above in section "Transaction signing and verification process", because of the uniqueness of the output of the ${\rho }_{\pi }^{+}(n)$ generating algorithm, we have $\left|{\rho }_{1}\right.\rangle =\left|{\rho }_{2}\right.\rangle$, and thus, the signatures cannot be forged.

In the second approach, a signer generates transaction information $TA1$ and public key $\left|PK\right.\rangle$. An attacker wants to forge the signer’s transaction information by turning it into $TA2$ $\ne TA1$ to make the signature of $TA2$ pass the verification process. According to the security of the private keys mentioned in section "Security of private keys", attackers have no way to generate a valid signature when they have no signers’ private keys. Therefore, transaction information cannot be forged. In Conclusion, the forging methods mentioned above cannot be performed.

#### Security against repudiation

Repudiation is that attackers repudiate signatures to make signers fail in the signing process.

According to the signature algorithm mentioned above in section "Transaction signing and verification process", an attacker has no access to verify the signatures when they are not a witness; hence, an attacker cannot repudiate signatures. When an attacker is a witness, Jack can automatically pass through the signature if verification succeeds. In this way, an attacker still cannot repudiate the signatures because Jack is a trusted node and determines whether a signature can pass through the verification process.

#### Security against interception

Interception is that attackers forge information through intercepting information.

According to the signature algorithm mentioned in section "Transaction signing and verification process", messages, including $TA,ID,\left|\rho {^{\prime}}\right.\rangle ,\left|PK{^{\prime}}\right.\rangle ,\left|\rho {^{\prime}}{^{\prime}}\right.\rangle ,\left|PK{^{\prime}}{^{\prime}}\right.\rangle$ and the location information of decoy particles, can be intercepted by an attacker.

In the signing phase,$TA,ID,\left|\rho {^{\prime}}\right.\rangle ,\left|PK{^{\prime}}\right.\rangle$ is first intercepted. Then, to avoid the suspects of the signer, the attacker has to forge a new message,$TA1,ID,\left|{\rho }_{1}\right.\rangle ,\left|PK{^{\prime}}\right.\rangle$, to pass the verification process, which is a man-in-the-middle attack. According to the analysis mentioned above in section "Security against forging", even if the attacker passes through the decoy particle check process, the forging messages still cannot pass through the verification process because the attacker has no signer’s private key.

In the verification phase, the attacker first intercepts $TA,ID,\left|\rho {^{\prime}}{^{\prime}}\right.\rangle ,\left|PK{^{\prime}}{^{\prime}}\right.\rangle$. Then, to avoid the suspicion of the signer, the attacker has to forge a new message $TA2,ID,\left|{\rho }_{2}\right.\rangle ,\left|PK{^{\prime}}{^{\prime}}\right.\rangle$ to pass through the verification process, which is a man-in-the-middle attack too. In this way, the reason for a forging failure is the same as that for a signing phase failure.

### Security issues from actual applications

In actual applications, there are several other security problems for businesses, organizations and operations. According to recent research progresses^27−30^, some kinds of techniques, such as Process-Data-Infrastructure (PDI) model^27^, can be incorporated into blockchain systems to figure out these problems and secure blockchain applications.

According to the PDI model^27^, system security issues can be classified to three levels: process level, data level and infrastructure level. The blockchain security in the process level includes operation standards, smart contracts, implementation security and fraud detection. The data level is composed of consensus algorithms, encryption, authentication, key management and access control while the infrastructure level includes super-node server, terminal devices and network. In the above sections we have discussed the blockchain security issues in the data level, and our blockchain can be combined with the modern blockchain frame (such as the PDI model) to enhance the security of the blockchain system. In blockchain-secured smart manufacturing ^28^, a specific PDI model can be realized like the following architecture: in the infrastructure level, a blockchain platform (such as Ethereum, Hyperledger, and EOS) is selected to manage terminals and networks. The platform should provide distributed data structure, interaction mechanisms, and computing paradigms. Then in the data level, our blockchain algorithm can be used to generate blocks (by consensus algorithm) and sign the transactions (by quantum digital signature) safely. More complex computations are performed safely with privacy computing (such as secured multi-party computation^24^, federated learning^31^ and trusted execution environment^32^), which makes blockchain compute functions on private data with them unexposed. Then a computer language supported by the blockchain platform is used to write smart contracts in the process level. Programmable manufacturing devices can be deployed in necessary places, and relevant data are collected through internet of things (IoT)^33^, which are transmitted to blockchain for next processing.

## Comparison with other quantum blockchain signature methods

In actual applications of blockchain technology, security is of the most importance; thus, we would use the safest signature algorithm as much as possible. Then, we will demonstrate that the signature algorithm in our blockchain algorithm is safer than other quantum-resistant signature algorithms. We assume that the decoherences of quantum circuits with outside environments can be ignored.

### Comparison with a signature algorithm based on nonorthogonal encoding

We introduce the basic ideas of nonorthogonal ^20^ encoding first. We define four quantum states: $\left|0\right.\rangle ,\left|1\right.\rangle ,\left|+\right.\rangle ,\left|-\right.\rangle$, where $\left|0\right.\rangle$ and $\left|1\right.\rangle$ are eigenstates of Pauli $Z$ and $\left|+\right.\rangle$ and $\left|-\right.\rangle$ eigenstates of Pauli $X$. The signer prepares a binary string $a=({a}_{1}{a}_{2}...{a}_{n}),{a}_{i}\in \{\mathrm{0,1}\}$, where $n$ is large enough, and selects a trusted authenticator. Then, we define four nonorthogonal sets, $\{|0\rangle ,|+\rangle \},\{|+\rangle ,|1\rangle \},\{|1\rangle ,|-\rangle \},\{|-\rangle ,|0\rangle \}$, and any two quantum states in each set are not orthogonal to each other. The signer, verifier and authenticator can perform the next procedures to complete this signature algorithm.

*Step 1* The signer selects a random quantum state from the four quantum states and distributes the nonorthogonal sets containing this quantum state according to the corresponding bit in the code. For example, if ${a}_{1}=1$ and the signer selects $\left|0\right.\rangle$, we distribute the set $\{|-\rangle ,|0\rangle \}$ to the first quantum state; if ${a}_{1}=0$ and the signer selects $\left|0\right.\rangle$, we distribute the set $\{|0\rangle ,|+\rangle \}$ to the first quantum state. This process is repeated $n$ times; then, the signer sends the quantum states $Q=({Q}_{1}{Q}_{2}...{Q}_{n}),{Q}_{i}\in \{|0\rangle ,|1\rangle ,|+\rangle ,|-\rangle ,\}$ and single bit information $m$ to the verifier and the authenticator by quantum channels.

*Step 2* The verifier and the authenticator choose an $X$ or $Z$ basis randomly for every quantum state ${Q}_{i}$ and then take the measurements of these quantum states ${\{Q}_{i}\}$.

*Step 3* The signer sends sets to the verifier and the authenticator by traditional channels.

*Step 4* The verifier and the authenticator compare every result of quantum state ${Q}_{i}$ with their sets. If one measurement result is orthogonal to one quantum state in the set, the conclusive bit ${a}_{i}$ can be obtained. For example, if we receive set $\{|0\rangle ,|+\rangle \}$ and the measurement result is $|-\rangle$, we can know that the signer sends 0; however, if the measurement result is not orthogonal to any quantum state in the set, the code the signer sent is inconclusive.

*Step 5* The signer sends a bit string $a$ to the verifier and authenticator. After receiving the bit string $a$, the verifier and the authenticator compare it with their conclusive bit string and compute the error rates $E(a{^{\prime}})$ and $E(a{^{\prime}}{^{\prime}})$, respectively (we take inconclusive bits as right bits). If both $E(a{^{\prime}})$ and $E(a{^{\prime}}{^{\prime}})$ are larger than threshold $\mu$, the signature fails; otherwise the signature can succeed.

It can be demonstrated that this signature algorithm cannot defend against interception. An attacker can perform the next procedures to forge a signer’s signature.

*Step 1* The signer generates single bit information $m$, bit string $a$ and quantum state $Q$ and then sends them to the verifier and the authenticator.

*Step 2* The attacker intercepts the messages $\{m,a,Q\}$; generates single bit information $m{^{\prime}}$, bit string $a{^{\prime}}$ and quantum state $Q{^{\prime}}$; and then sends $\{m{^{\prime}},a{^{\prime}},Q{^{\prime}}\}$ to the verifier and the authenticator.

*Step 3* The signer sends sets $Q1$ of $Q$ to the verifier and the authenticator.

*Step 4* The attacker incepts messages $Q1$ and sends sets $Q2$ of $Q{^{\prime}}$ to the verifier and the authenticator.

*Step 5* The verifier and the authenticator perform step 5 in the signature algorithm.

*Step 6* Now, the attacker forges a perfect signature of the signer because it is simple to generate $\{m{^{\prime}},a{^{\prime}},Q{^{\prime}}\}$ and $Q2$, so the verifier and the authenticator can pass the signature forged by the attacker with overwhelming probability. A forging attack can work in this way.

As mentioned in section "Security against interception", we have demonstrated that the signature algorithm in our blockchain algorithm can resist interception and thus is safer than the algorithm in this section.

### Comparison with a signature algorithm based on quantum entanglement

Suppose there are three characters that take part in this algorithm ^16^: the signer, the verifier and a trusted node blockchain. They perform the next procedures to complete this signature algorithm.

*Step 1* The blockchain generates sufficient Bell states: $\{({A}_{1}^{1},{A}_{1}^{2}),({A}_{2}^{1},{A}_{2}^{2}),...,({A}_{n}^{1},{A}_{n}^{2})\}$, where $({A}_{i}^{1},{A}_{i}^{2})$ represents Bell state $|\psi {\rangle }_{{A}_{i}^{1}{A}_{i}^{2}}=\frac{|00\rangle +|11\rangle }{\sqrt{2}}$. Hence, we have two qubit strings $({A}_{1}^{1},{A}_{2}^{1},...,{A}_{n}^{1})$ and $({A}_{1}^{2},{A}_{2}^{2},...,{A}_{n}^{2})$. In the same way, the blockchain generates Bell states: $\{({B}_{1}^{1},{B}_{1}^{2}),({B}_{2}^{1},{B}_{2}^{2}),...,({B}_{n}^{1},{B}_{n}^{2})\}$, so we have two qubit strings $({B}_{1}^{1},{B}_{2}^{1},...,{B}_{n}^{1})$ and $({B}_{1}^{2},{B}_{2}^{2},...,{B}_{n}^{2})$.

*Step 2* The blockchain randomly selects a sufficiently long substring ${A}_{1}$ from $({A}_{1}^{1},{A}_{2}^{1},...,{A}_{n}^{1})$ and sends it to the signer as his private key; the blockchain randomly selects a sufficiently long substring ${A}_{2}$ from $({A}_{1}^{2},{A}_{2}^{2},...,{A}_{n}^{2})$ and sends it to the verifier as the signer’s private key; the blockchain randomly selects a sufficiently long substring ${B}_{1}$ from $({B}_{1}^{1},{B}_{2}^{1},...,{B}_{n}^{1})$ and sends it to the verifier as the private key; the blockchain randomly selects a sufficiently long substring ${B}_{2}$ from $({B}_{1}^{2},{B}_{2}^{2},...,{B}_{n}^{2})$ and sends it to the signer as the verifier’s private key.

*Step 3* The signer uses the hash function ($h=hash(m)$) on an x-length quantum coin $m=\left\{{m}_{1},{m}_{2},\dots ,{m}_{x}\right\},{m}_{i}\in \{|0\rangle ,|1\rangle \}$ to obtain a y-length hash sequence.

*Step 4* The signer performs controlled-NOT(CNOT) on the first x qubits of ${B}_{2}$, the first y qubits of ${A}_{1}$, quantum coin $m$ and hash sequence $h$:

$$CNO{T}_{{B}_{2i}{m}_{i}}|\psi {\rangle }_{{B}_{2i}{B}_{1i}}{m}_{i}=\frac{\left|00\right.\rangle {m}_{i}+\left|11\right.\rangle {\overline{m} }_{i}}{\sqrt{2}},$$

$$CNO{T}_{{A}_{1i}{h}_{i}}|\psi {\rangle }_{{A}_{2i}{A}_{1i}}{h}_{i}=\frac{\left|00\right.\rangle {h}_{i}+\left|11\right.\rangle {\overline{h} }_{i}}{\sqrt{2}},$$

where ${\overline{m} }_{i}=1-{m}_{i}\mathrm{ and }{\overline{h} }_{i}=1-{h}_{i}$. Then, the signer obtains quantum coin $m{^{\prime}}$ and hash sequence $h{^{\prime}}$ and sends $m{^{\prime}}$ and $h{^{\prime}}$ to the verifier.

*Step 5* The verifier performs CNOT on the first x qubits of ${B}_{1}$, the first y qubits of ${A}_{2}$, quantum coin $m$ and hash sequence $h{^{\prime}}$:

$$CNO{T}_{{B}_{1i}{m}_{i}}\frac{\left|00\right.\rangle {m}_{i}+\left|11\right.\rangle {\overline{m} }_{i}}{\sqrt{2}}=|\psi {\rangle }_{{B}_{2i}{B}_{1i}}{m}_{i},$$

$$CNO{T}_{{A}_{2i}{h}_{i}}\frac{\left|00\right.\rangle {h}_{i}+\left|11\right.\rangle {\overline{h} }_{i}}{\sqrt{2}}=|\psi {\rangle }_{{A}_{2i}{A}_{1i}}{h}_{i},$$

Then, the verifier obtains quantum coin $m{^{\prime}}{^{\prime}}$ and hash sequence $h{^{\prime}}{^{\prime}}$, computes $hash(m{^{\prime}}{^{\prime}})$ and judges if it is equal to $h{^{\prime}}{^{\prime}}$. If $hash(m{^{\prime}}{^{\prime}})=h{^{\prime}}{^{\prime}}$, the signature is accepted; otherwise, the signature is rejected.

It can also be demonstrated that this signature algorithm cannot defend against interception. An attacker can perform the next procedures to forge a signer’s signature.

*Step 1* As shown in the signature algorithm mentioned above, the blockchain generates substrings ${A}_{1}$, ${A}_{2}$, ${B}_{1}$, and ${B}_{2}$ and sends ${A}_{1}$ and ${B}_{2}$ to the signer and ${A}_{2}$ and ${B}_{1}$ to the verifier.

*Step 2* The attacker intercepts ${A}_{2}$ and ${B}_{1}$ through a man-in-the-middle attack, imitates the blockchain to generate substrings ${C}_{1}$, ${C}_{2}$, ${D}_{1}$, and ${D}_{2}$, retains substrings ${C}_{1}$ and ${D}_{2}$, and then sends substrings ${C}_{2}$ and ${D}_{1}$ to the verifier.

*Step 3* In this moment, the attacker’s substrings entangle the signer’s and the verifier’s at the same time, so the attacker can forge any transaction messages and the signatures of the signer and the verifier.

## Conclusion

We propose a quantum blockchain algorithm that generates blocks by DPoSB and signs the transaction information with a quantum one-way function based on the ${\text{QSC}}{\text{D}}_{\text{ff}}$ problem. By the stake vote and punishing the malicious behaviours of DPoSB and asymmetric quantum encryption, the fairness, efficiency and security of the blockchain system can be improved. Security in the semi-honest adversary model and the malicious adversary model can be realized in our blockchain based on quantum information-theoretical security. Furthermore, we demonstrate the security of our blockchain algorithm compared with other quantum blockchain algorithms. Our quantum blockchains provide a safe platform that could decrease the costs of various operations and transaction activities. We should mention that the trusted node used in our blockchain has a larger weight in the network and therefore the necessity of the trusted node may weaken decentralization. Quantum signatures which do not require the trusted node could be developed in future researches to solve this problem. Moreover, quantum blockchains could be based on quantum privacy computing, which would further enhance the security of actual blockchain applications. In the near future, quantum blockchains will play an important role in social and financial areas that have increasing demands for transaction securities.

## Data Availability

The authors declare that the data that support the plots within this paper and other findings of this study are available from the corresponding author upon reasonable request.
